# *Leptospira interrogans* Outer Membrane Protein-Based Nanohybrid Sensor for the Diagnosis of Leptospirosis

**DOI:** 10.3390/s21072552

**Published:** 2021-04-06

**Authors:** Vivek Verma, Deepak Kala, Shagun Gupta, Harsh Kumar, Ankur Kaushal, Kamil Kuča, Natália Cruz-Martins, Dinesh Kumar

**Affiliations:** 1 School of Bioengineering and Food Technology, Shoolini University, Solan 173229, HP, India; vivek77verma@gmail.com (V.V.); microharshs@gmail.com (H.K.); 2 Amity Center of Nanotechnology, Amity University, Gurugram 122413, HR, India; deepak.kala@student.amity.edu; 3 College of Horticulture & Forestry-Neri, Hamirpur 177001, HP, India; shagun22_88@yahoo.com; 4 Center for Basic and Applied Research, Faculty of Informatics and Management, University of Hradec Kralove, 50003 Hradec Kralove, Czech Republic; kamil.kuca@uhk.cz; 5 Faculty of Medicine, University of Porto, Alameda Prof. Hernâni Monteiro, 4200-319 Porto, Portugal; 6 Institute for Research and Innovation in Health (i3S), University of Porto, 4200-135 Porto, Portugal; 7 Laboratory of Neuropsychophysiology, Faculty of Psychology and Education Sciences, University of Porto, 4200-135 Porto, Portugal

**Keywords:** DNA sensor, *Leptospira interrogans*, *Loa22* gene, AuN/CNF

## Abstract

Leptospirosis is an underestimated tropical disease caused by the pathogenic *Leptospira* species and responsible for several serious health problems. Here, we aimed to develop an ultrasensitive DNA biosensor for the rapid and on-site detection of the *Loa22* gene of *Leptospira interrogans* using a gold nanoparticle–carbon nanofiber composite (AuN/CNF)-based screen-printed electrode. Cyclic voltammetry and electrochemical impedance were performed for electrochemical analysis. The sensitivity of the sensor was 5431.74 μA/cm^2^/ng with a LOD (detection limit) of 0.0077 ng/μL using cyclic voltammetry. The developed DNA biosensor was found highly specific to the *Loa22* gene of *L. interrogans*, with a storage stability at 4 °C for 180 days and a 6% loss of the initial response. This DNA-based sensor only takes 30 min for rapid detection of the pathogen, with a higher specificity and sensitivity. The promising results obtained suggest the application of the developed sensor as a point of care device for the diagnosis of leptospirosis.

## 1. Introduction

Leptospirosis is an acute bacterial septicemic disease caused by the pathogenic spirochaetes of the genus *Leptospira* [1]. In humans, leptospirosis has biphasic (anicteric and icteric) clinical manifestations that include flu-like symptoms in the anicteric phase and mild infection to multiple organ failure (Weil’s syndrome) in the icteric phase [2]. Laboratory-based tests are the most important and effective way to diagnose the disease, as its signs and symptoms are very common and similar to other diseases/disorders. Several methods are available for the detection of leptospirosis that include polymerase chain reaction (PCR), multiplex loop-mediated isothermal amplification (m-LAMP), Immunoglobulin-M (IgM)-based enzyme-linked immunosorbent assay (IgM ELISA), and the microscopic agglutination test (MAT) [3,4]. Among all the above-mentioned, the MAT is considered the gold standard test for the detection of *Leptospira serovars* [5]. However, these traditional methods are time-consuming, expensive, and not able to lay bare the infection at the early stages.

Molecular diagnosis is an advanced way to detect the disease more precisely and rapidly in comparison to the traditional methods [6,7,8]. These molecular diagnosis methods include PCR, LAMP, a DNA biosensor that targets specific genetic markers in the stretch of leptospiral single stranded genomic DNA (ssGDNA) isolated from the patient’s blood/cerebrospinal fluid (CSF)/urine samples [9,10]. Most molecular assays are developed either by targeting the housekeeping genes such as *gyrB*, *sec Y*, or *rrs* or by species-specific pathogenic genes like *lfb1, lipL32, ligB*, or *Loa22* [11,12].

In this sense, novel detection methods that provide ease, high sensitivity, and speed are still very much needed in the basic medical diagnosis and control of the disease. There is a need to devise a faster, more accurate, sharper, and less expensive method of *L. interrogans* detection [13,14,15]. The emerging diagnostic methods, such as biosensors, are in demand because of their better performance in terms of onsite detection ability with less time and higher clinical sensitivity. An electrochemical DNA biosensor is becoming a need of ours due to its ability to lay bare the infection at the early stages with better sensitivity and accuracy [16,17,18,19,20,21]. It can create a point-of-care diagnostics facility even outside the laboratory settings and can be a replacement of the current diagnosis methods that require sophisticated instrument facilities and experts to carry out the tests [22]. In the way of such advancements, a step has already been taken by Nagraik et al. by developing an amperometric DNA sensor for the diagnosis of leptospirosis by targeting the highly conserved *LipL32* gene of *L. interrogans* as a genetic marker [23]. The sensor was reported with higher sensitivity, specificity, and storage stability. 

Pathogenic genes are highly specific and useful in the identification of virulent strains. Among all surface protein-encoding genes, *Loa22* is the only gene that fulfills Koch’s molecular postulates for virulent factors. The *Loa22* gene encodes a 22-kDa lipoprotein on the cell surface and contains a C-terminal OmpA domain. *Loa22* is a virulent gene that helps in the attachment of bacteria to the host cell surface and helps in its penetration [12]. Thus, the present study was focused on the fabrication of a *Loa22* gene-based amperometric DNA biosensor for the detection of *L. interrogans* to improve the sensitivity, selectivity, and specificity of the diagnosis method. The sensor was constructed using a screen-printed electrode consisting of gold-embedded carboxylated carbon nanofiber (AuN/c-CNFs). The physical and chemical properties of carbon nanofibers (CNFs) make them a suitable nanomaterial for novel composites that can enhance the properties of composite materials due to the synergistic interactions. The graphitic and edge planes of CNFs have the potential for surface functionalization or modifications that can be useful in surface modifications for the binding of the bioreceptors [16] and also has great potential for biosensing applications due to the larger surface area, high conductivity, and electrocatalytic activity [24].

## 2. Materials and Methods

### 2.1. Materials

Methylene blue (MB), Tris ethylenediamine-tetraacetic acid (EDTA), Sodium chloride (NaCl) from Himedia, and ethanol (C_2_H_5_OH) were obtained from Chanshu Hong sheng Fine Chemical Co. Ltd. Disodium hydrogen orthophosphate (Na_2_HPO_4_), sodium di-hydrogen orthophosphate (NaH_2_PO_4_), hydrochloric acid (HCl), and other chemicals were from Qualigens, India. 1-ethyl-3-(3-dimethylamino propyl)-carbodiimide (EDC) and N-hydroxysuccinimide (NHS) were purchased from Sigma-Aldrich, USA. Electrodes used in the experiment (AuN/c-CNFs) were from DropSens, Spain. Bacterial samples were collected and processed in the Post Graduate Institute of Medical Education and Research (PGIMER), Chandigarh. The *Loa22* gene specific amine-labeled ssDNA probe (5′NH2-TCCCGAACAAGCAGAAGGTG-3′) was synthesized from Eurofins Genomics India Pvt. Ltd.

### 2.2. Equipment

The Palmsens 4 model of Potentiostat/Galvanostat was used for the electrochemical studies. Screen-printed AuN/CNF electrodes (DropSens, Spain) were purchased and modified at Shoolini University, Solan (H.P.). Nanodrop spectrophotometer (QIAXPERT, QIAGEN, Germany) was used for the qualitative and quantitative analyses of DNA. The Perkin Elmer Fourier-transform Infrared spectrometer and ANDOR SR-500i-B2 model Raman spectrophotometer were used (656-nm wavelengths). 

### 2.3. Isolation of Genomic DNA 

Patient’s sample processing and the ssGDNA isolation were performed at the Department of Microbiology, PGIMER, Chandigarh, India using the method described by Pereira et al. [25]. The isolated GDNA was further processed (denatured at 95 °C for 5 min to convert it into the ssDNA form for hybridization) for analysis using the developed DNA sensor [26].

### 2.4. Fabrication of the AuN/CNFs DNA Sensor

**Scheme 1:**
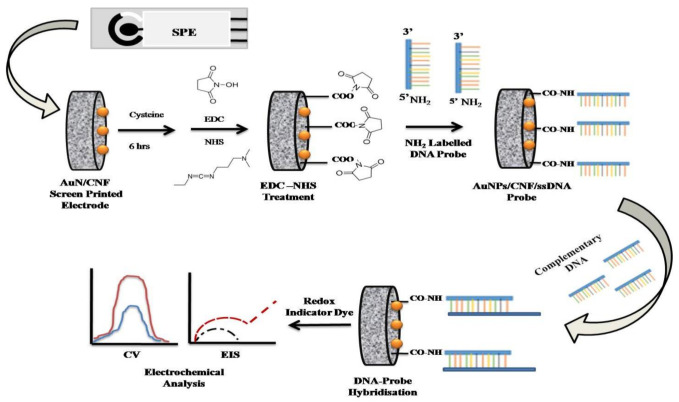
Illustration of steps involved in the construction of the DNA sensor. AuN: Gold Nanoparticles, CNF: Carbon Nanofiber, SPE: Screen-Printed Electrode, MPA: 3-Mercaptopropionic acid, CV: Cyclic Voltammetry, EIS: Electrochemical Impedance Spectroscopy, EDC: 1-Ethyl-3-(3-dimethyl aminopropyl) carbodiimide, and NHS: N-Hydroxysuccinimide.

AuN/c-CNF screen-printed electrodes were used for the construction of the *Loa22* gene-based DNA sensor. The 6 μL volume of 5mM Cysteine (Cys) solution was added on the surface of a working electrode (0.126 cm^2^) and incubated overnight for adherence to Au nanoparticles. The incubation step was followed by the multiple washing steps using double-distilled water to remove the excess of cysteine from the surface. The equimolar amount (1 mM) of 1-ethyl-3-(3-dimethylamino propyl)-carbodiimide (EDC) and N-hydroxysuccinimide (NHS) in a *v*/*v* ratio of 1:1 in phosphate-buffered saline (PBS) (pH 7.2) was added on an electrode surface for 2 h to trigger the carboxyl group (-COOH) for carbodiimide crosslinking. The electrode was further washed multiple times using phosphate buffer (PBS, pH 7.2) and dried at room temperature (RT). The amine-linked (5′ NH_2_) ssDNA probe (6 μL, 10 μM) was added on an activated working electrode surface (AuN/c-CNFs) and incubated in a humid chamber for 5 h. After incubation, the ssDNA probe-modified electrode (AuN/c-CNFs/ssDNA_probe_) was washed 2 to 3 times using Tris-EDTA buffer (TE) (pH 8.1) to eliminate the unbounded probe. The leptospiral genomic DNA (denatured at 95 °C/5 min) was used for hybridization with the electrode (AuN/c-CNFs/ssDNA_probe_) for 15 min at RT. The above-mentioned step was repeated for hybridization with different dilutions of the *L. interrogans* ssGDNA. The cyclic voltammetry (CV) analysis was performed using Galvanostat/Potentiostat at a potential scan of −1 × 10^3^ to 5 × 10^2^ mV using methylene blue (1-mM PBS, pH 7.2). The electrochemical impedance was recorded using 1-mM potassium ferricyanide at a frequency scan of 10^−2^–10^5^ Hz. The fabrication steps involved in the construction of the DNA sensor are illustrated in Scheme 1.

### 2.5. Selectivity of the Sensor

The selectivity of the DNA sensor against the complementary DNA sequence was evaluated by inducing a different number of base mismatches in the complementary DNA (cDNA) sequence (Table 1). The selectivity of the sensor was calculated in terms of the relative peak current *Ip* obtained before and after hybridization of the DNA sensor with cDNA and mismatched cDNA sequences. The percent peak current value (% *Ip*) was calculated using the following equation:

(1) $$\%Ip=(\frac{Ipnc\mathrm{DNA}}{Ipc\mathrm{DNA}})\times 100\%$$

where *Ip*cDNA and *Ipnc*DNA represents the complementary and noncomplementary (mismatched DNA base) DNA sequences, respectively.

## 3. Results and Discussion

### 3.1. Characterization Study

**Figure 1:**
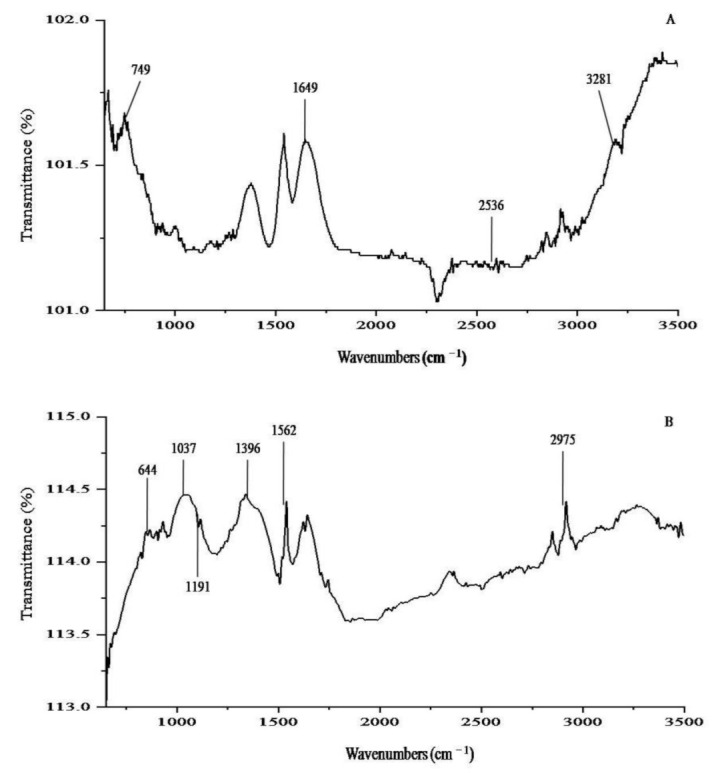
Characterization of the DNA sensor fabrication steps using a Fourier-transform infrared spectroscopy (FTIR) analysis of (**A**) the AuN/CNF bare electrode and (**B**) after modifications of the AuN/CNF electrode surface with a single stranded DNA (ssDNA) probe (AuN/CNF/ssDNA_probe_) at a frequency scan of 500–3500 cm^−1^.

The electrode was characterized at a different phase of the sensor development, including the bare electrode and ssDNA probe-modified electrode, and after hybridization of the DNA sensor (DNA Probe) with ssGDNA of *L. interrogans.* Fourier-transform infrared spectroscopy (FTIR) and Raman spectroscopy were carried out to ensure each step of the DNA sensor development process [16]. FTIR studies revealed the functional groups imparted on the working electrode (AuN/CNF) after the modification with the ssDNA_(probe)_ (Figure 1). 

**Figure 2:**
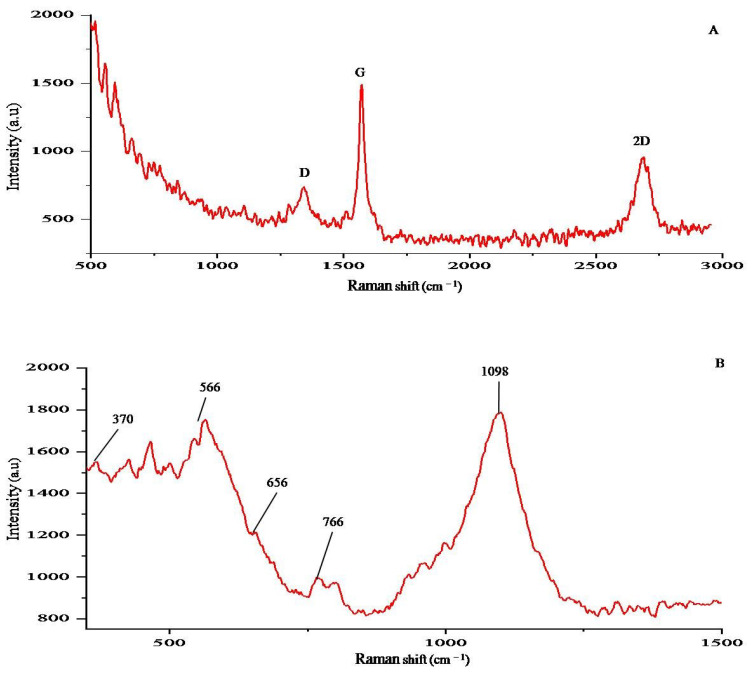
Raman spectrum of (**A**) the bare AuN/CNF electrode and (**B**) 5′amino-linked ssDNA probe-modified AuN/CNF electrode (AuN/CNF/ssDNA_(probe)_).

The comparison of the DNA sensor development phases from the bare AuN/CNF/Cys (Figure 1A) electrode to AuN/CNF/ssDNA_probe_ (Figure 1B) showed transmittance peaks across, 644, 1037, 1191, and 2975 cm^−1^. The transmittance peaks of the bare electrode across 749, 1649, and 2536 cm^−1^ is of C-H, C=O, and a simple O-H, respectively. The ssDNA probe-modified electrode showed the transmittance peaks at 644, 1037, and 1191 cm^−1^ corresponding to thymine, guanine, cytosine, and adenine (TGCA), respectively, that ensures the existence of DNA bases on the electrode surface. Further, the transmittance peak at 2975 cm^−1^(PO^2−^) is a characteristic peak of the DNA-phosphate backbone that supports the above data and ensures the immobilization of the ssDNA probe onto the working electrode surface. The phases of the DNA sensor development were also studied using Raman spectroscopy. The Raman spectra of the ssDNA probe-modified electrode shows across 370 and 566 cm^−1^ (vibration of A); 656 cm^−1^ (vibration of A, C, and T); 766 cm^−1^ (vibration of C); and 1098 cm^−1^ (PO^2−^ vibration) that confirmed the presence of ssDNA _probe_ on the bare electrode (Figure 2). Raman spectrum supported the results obtained from the FTIR spectrum and ensured the different steps of the DNA sensor development process.

### 3.2. Electrochemical Analysis

**Figure 3:**
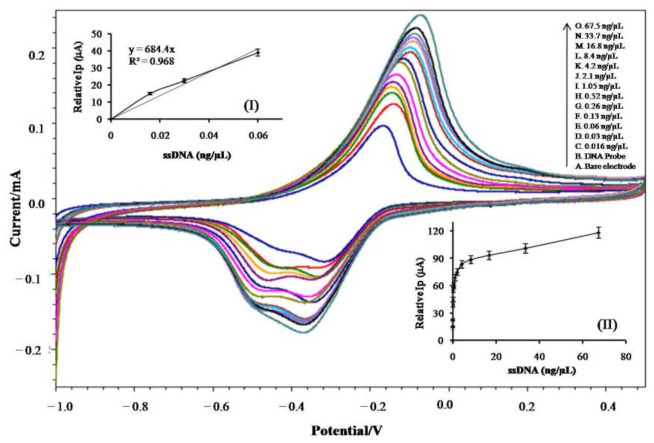
Voltammetric analysis of the developed DNA sensor in different phases of the fabrication, including (A) the AuN/CNF electrode (bare), (B) AuN/CNF/ssDNA (probe), and (C–O) hybridization with single-stranded GDNA of *Leptospira interrogans*. The insert (**I**) shows a linear curve for the calculation of the limit of detection (LOD) and (**II**) shows a hyperbolic curve plotted between the relative peak current *Ip* with respect to probe with different concentrations of hybridizing ssGDNA of *L. interrogans*.

**Figure 4:**
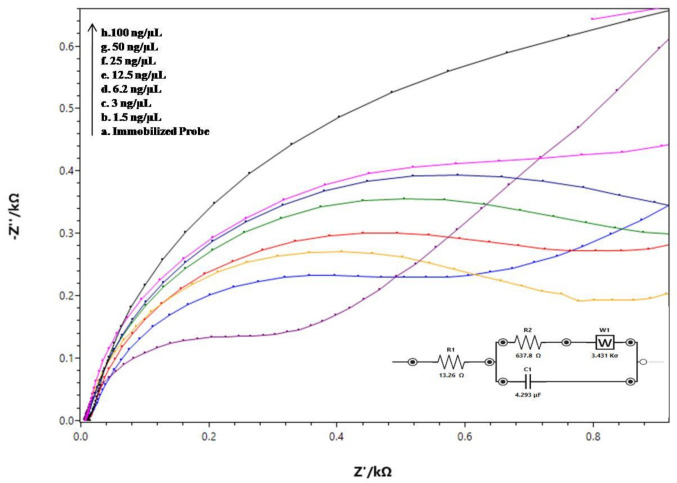
Comparison of the electrochemical impedance spectra of the DNA chip fabrication steps, including (a) the immobilization of the 5′amino-linked DNA probe and (b–h) hybridization with various concentrations of ssDNA of *L. interrogans* using 1-mM Potassium ferricyanide solution.

The DNA sensor response in terms of the peak current (*Ip*) and regression coefficient (R^2^_t_) was measured using CV (Figure 3) and electrochemical impedance spectroscopy (Figure 4), respectively. 

The sensor response (*Ip* value) was changed (increased) after the hybridization with ssGDNA and raised similarly with different concentrations of *L. interrogans* ssGDNA (0.016–67.5 ng/µL). The plot of the *Ip* values vs. concentration of the ssGDNA of *L. interrogans* was hyperbolic and showed a linear response in between 0 to 0.52 ng/µL with a regression coefficient (R^2^) of 0.968. The DNA sensor sensitivity, i.e., 5431.74-(µA/cm^2^)/ng DNA was calculated using the response (*Ip*) of the DNA sensor with different concentrations of ssGDNA of *L. interrogans.* The limit of detection (LOD) of the DNA sensor was determined as 0.0077 ng/µL, which was in agreement with the experimental value (minimum ssGDNA concentration). An increase in the *Ip* value with different concentrations of *L. interrogans* GDNA was due to the presence of guanine bases on the DNA sensor surface, which showed a binding affinity with methylene blue (MB) [16].

The increase in the *Ip* value with the concentrations of *L. interrogans* ssGDNA was due to the increase in the DNA bases (guanine) on the electrode surface, which provides more binding sites for MB and increases the current response during the electrochemical measurement using CV. The DNA sensor response was also recorded by electrochemical impedance at a frequency scan of 10^−2^–10^5^ Hz using 1 mM-K_3_Fe(CN)_6_ in 1-mM PBS buffer (pH 7.2). The charge transfer resistance (R_ct_) was calculated to confirm the fixation of the ssDNA_(probe)_ onto the AuN/CNF bare electrode and its hybridization with the ssDNA of *L. interrogans.* The recorded values show the higher R_ct_ value of AuN/CNF/ssDNA (probe) in comparison to the AuN/CNF bare electrode. The raise in the R_ct_ value of the AuN/CNF/ssDNA (probe) was due to the presence of the ssDNA negatively charged phosphate backbone, which resists the [Fe(CN)_6_]^3−^ ions to reach the electrode surface (AuN/CNF) (Figure 3). Further, the DNA sensor hybridization with various concentrations of single-stranded (ss)GDNA of *L. interrogans* revealed an increase in the R_ct_ values due to the enrichment of the electrode surface with nucleotide bases, causing more repulsion of the [Fe(CN)_6_] ions. The obtained pattern of impedance ensures the immobilization of the ssDNA probe onto the AuN/CNF electrode surface and its hybridization with the ssGDNA of *L. interrogans.*

The developed assay was found to be more specific and sensitive than the previously reported biosensors [23,27] developed for detecting leptospirosis. The performance of the electrochemical DNA biosensor depends upon the surface area and conductivity of the working electrode surface. Different types of carbon nanomaterials and nanocomposites have been used for the construction of biosensors for the detection of leptospirosis (Table 2), and out of this, an AuN/CNF-based biosensor was found to be better in terms of the sensitivity, selectivity, specificity, and response time.

### 3.3. Selectivity of the Sensor

**Figure 5:**
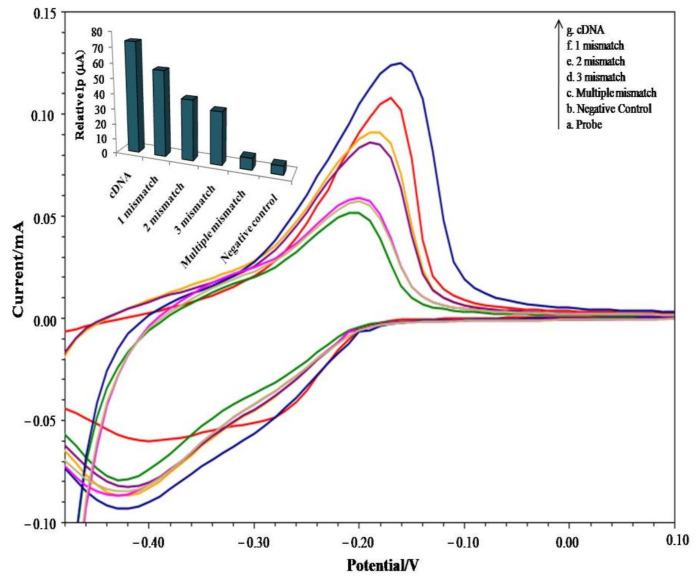
Cyclic voltametric analysis of DNA sensor selectivity using cDNA (complementary DNA) and a sequence with different numbers of mismatched bases. The insert shows the relative peak current values % *Ip* (with respect to the probe) of the DNA sensor with cDNA and different numbers of mismatched bases.

The sensor selectivity was evaluated using the cDNA and its sequence with a different number of mismatched DNA bases (Figure 5). 

The sensor was found highly selective to the complementary DNA sequence, as the maximum response (% current value) was obtained with it, and the values decreased with increasing the number of mismatched bases, i.e., one base, two bases, three bases, and multiple bases in the cDNA sequence. The multiple bases mismatched sequence even shows the current response equivalent to the probe that ensures a very low response of the sensor towards the mismatched sequences. The sensor response with cDNA and mismatched DNA sequences suggested its selectivity towards the targeted sequence (cDNA). The inconsiderable current response of the DNA sensor with different numbers of mismatched bases authenticates its ability to discriminate between the complete cDNA sequences with the mutated sequences.

### 3.4. Specificity Tests

**Figure 6:**
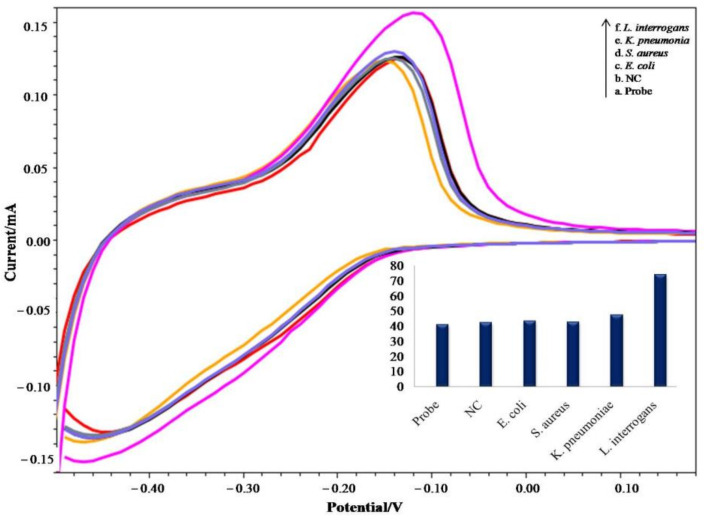
Evaluation of the DNA sensor specificity with *L. interrogans* and other bacterial species (*Escherichia coli, Staphylococcus aureus*, and *Klebsiella pneumoniae*) using cyclic voltametric studies (NC= Negative Control). The insert shows the relative *Ip* values (with respect to the probe) of the DNA sensor with the hybridizing GDNA of *L. interrogans* and other bacteria.

The sensor was found highly specific for leptospirosis (Figure 6), as the only hike in peak current (*Ip*) value was observed with the ssGDNA of *L. interrogans*, and no significant changes in the *Ip* values were recorded with the ssGDNA of other bacteria (*S. aureus, E. coli*, and *K. pneumoniae*). The DNA sensor response only to the targeted organism proved its specificity towards the desired analyte, and no cross-reactivity was observed with other bacterial ssGDNA.

## 4. Conclusions

The developed DNA sensor was revealed to be better than the previously reported methods in terms of the sensitivity, selectivity, specificity, and LOD. The sensor showed a sensitivity of 5431.74 μA/cm^2^/ng, which was 20 times higher than the previously reported DNA sensor. The LOD, i.e., 0.0077 ng/μL was also better than the previously reported methods. The sensor was found highly selective and specific for the target DNA sequence, as a higher current response in cyclic voltammetry was observed only with the targeted ssGDNA (*L. interrogans*). The sensor specificity towards *L. interrogans* genomic DNA enabled it to discriminate between the target DNA sequences with other possible contaminations (bacterial ssGDNA) in patients’ DNA samples. In terms of storage stability, the developed DNA sensor revealed a valid response if stored at 4 °C for six months, as indicated by the CV analysis. The compact design and portability of the developed DNA sensor make it an idle point of care POC device for monitoring infections in rural areas within a short period.

## Figures and Tables

**Table 1:** The nucleotide sequence of the 5′amino-linked single stranded ssDNA probe, its complementary DNA, and mismatched DNAs.

DNA Sample	Mismatch Base Sequences
DNA Probe (AmC6)	5′TCCCGAACAAGCAGAAGGTG3′
cDNA	5′CACCTTCTGCTTGTTCGGGA3′
1 BMM	5′TACCTTCTGCTTGTTCGGGA3′
2 BMM	5′TGCCTTCTGCTTGTTCGGGA3′
3 BMM	5′TGACTTCTGCTTGTTCGGGA3′
MBMM	5′TGAGTTATACTTGGGTGGTC3′

cDNA: Complementary DNA, BMM: base mismatch, and MBMM: Multiple base mismatches.

**Table 2:** Comparison of the different types of biosensors developed for the detection of leptospirosis with the present method based on the *Loa22* gene of *Leptospira interrogans.*

SN	Type of Sensor	Biomarker	Nanomaterials	Sensitivity	LOD/ * LOQ	Linearity	Ref.
1	Amperometric DNA sensor	*LipL32* gene	AuN/cMWCNT	264.5 μA/cm^2^/ng	0.015 ng/6 μL	ND	[23]
2	Electrochemical immunosensor	*rLipL32* protein	SPGE	ND	0.28 ng/mL * 0.93 ng/mL	1 to 100 ng/mL	[28]
3	Amperometric DNA sensor	*LipL32* gene	AuN/cMWCNT	ND	ND	0.37 to 12 ng/μL	[27]
4	Amperometric DNA sensor	*Loa22* gene	AuN/CNF	5431.74 μA/cm^2^/ng	0.0077 ng/μL	0.016 to 67.5 ng/µL	PM

AuN: Gold nanoparticles, cMWCNT: carboxylated multi-walled carbon nanotubes, SPGE: screen-printed graphene electrode, LOD: limit of detection, LOQ: limit of quantification, rLipL32: recombinant LipL32, ND: not defined, and PM: present method. * LOQ value sign was used with LOQ to differentiate it from LOD values in the table.

## Data Availability

Not applicable.
